# Screen time and adolescent well-being: a comparative study of Russia and Cuba

**DOI:** 10.3389/fpubh.2025.1587475

**Published:** 2025-08-05

**Authors:** Dmitry Kornienko, Natalia A. Rudnova, Aleksander Veraksa, Jorge Enrique Torralbas Oslé, Apollinaria Chursina, Emely Corcho Rosales

**Affiliations:** ^1^FSBSI Federal Scientific Center of Psychological and Multidisciplinary Research (FSC PMR), Moscow, Russia; ^2^Facultad de Psicología de la Universidad de La Habana, La Habana, Cuba

**Keywords:** screen time, adolescents, social media addiction, health, well-being, cross-cultural comparison, internet usage, social media

## Abstract

**Introduction:**

The increasing prevalence of devices with unrestricted internet connectivity among the younger population gives rise to a novel environment for social interaction and cognitive processing. Most research have concentrated on adolescents in the Western countries, addressing the influence of screen time on their social and cognitive growth. The objective of the study was to examine the association between the amount of time spent on screens and the presence of health and well-being problems among adolescents from Russia and Cuba. Considering the prevailing worldwide pattern of excessive internet usage and social media consumption, we expect to observe rare and minor variations in screen time and its effects on health and well-being.

**Methods:**

The sample consists of 524 adolescents between the ages of 13 and 18 (*M* = 15.6; SD = 1.28), with 58.7% female. The study included 224 people residing in Cuba and 300 participants residing in Russia. The online survey comprises inquiries regarding internet and gadget usage duration, addiction to social media, positive and negative affects, and items for evaluating screen time induced problems, and preferred content.

**Results:**

There are significant differences in the amount of time spent on screens, addiction to social media, overall well-being, and health issues among adolescents from Russia and Cuba. Adolescents in Russia exhibited elevated levels of internet usage and engagement on social media platforms; however, they experienced an increase in cognitive and family-related issues. Cuban adolescents, in turn, experience more negative affect and problems with basic needs due to excessive Internet use.

**Conclusion:**

Generally, irrespective of their country of residence, teenagers who spend more time on screens tend to encounter a higher prevalence of health and well-being problems. The environment of Russian adolescents is significantly more like European countries in terms of Internet use, which has led to an increase in screen time and various health and psychological issues. In contrast, Cuban teenagers experience increased issues with family relationships because of screen time. The findings are consistent with prior research that have shown a correlation between increased internet usage and adverse outcomes.

## 1 Introduction

Screen time has been under increasing scrutiny due to its potential effects on the health and well-being of children and adolescents. Research has indicated that excessive use of digital devices may be associated with adverse consequences for mental and physical health, including increased anxiety, depression, and social development issues (1, 2).

Various studies have documented negative effects of screen time on physical health, such as increased risk of obesity, poor dietary habits and physical inactivity (3, 4). Studies have shown an association between increased screen time and depressive symptoms in children and adolescents (2, 5). However, despite the replicable results, the effect sizes are often modest or even small, and other variables like socio-demographics (i.e., socioeconomic status) or type of device and usage practices could influence more (2).

The substantial increase in screen time exposure during the COVID-19 pandemic has been associated with a corresponding rise in social media addiction among adolescent populations (57, 6). Social media addiction can be conceptualized through Griffiths’ (7) biopsychosocial model of addiction, which identifies six core components of addictive behavior: salience, mood modification, tolerance, withdrawal symptoms, relapse, and conflict. This framework has been empirically validated within digital contexts through Kuss and Griffiths’ (8) internet addiction components model, demonstrating its applicability to problematic social media use. Adolescents who spend more time on social media are at higher risk for developing addictive patterns of social media use, which are linked to negative mental health outcomes such as depression or even a self-harm (9, 10). Screen time duration is not a direct indicator of problematic social media because the relationship between them is influenced by individual and contextual factors, including emotional well-being, sensitivity to social feedback, being influenced by influencers, and socio-economic status (11, 12). While excessive screen time is linked to negative outcomes, moderate use, especially when balanced with physical activity, can be associated with positive mental health outcomes. For instance, higher phone use was linked to higher positive mental health when combined with physical activity (13). Additionally, participation in extracurricular activities alongside limited screen time is associated with higher life satisfaction and optimism (58).

### 1.1 Screen content and mental health in children

The relationship between screen content and mental health in children is complex and varies depending on the type of content consumed. Overall, both the amount of screen time and the type of content can impact children’s mental health. This association is more pronounced with longer durations of screen use, particularly for recreational activities like watching TV (58, 2, 13, 14).

One of the most popular types of content among young people is entertainment and gaming, which mostly has a detrimental effect on mental health, particularly when consumed in large amounts (15). Social media use also occupies a significant portion of screen time. Studies often highlight the negative effects of the use of social media and interactive media, which is consistently associated with poorer mental health outcomes (16).

Social media plays a multifaceted role in adolescents’ lives, impacting their mental health, development, and social interactions. Due to the pervasive presence of social media, they provide support, connection, and self-expression fulfilling basic psychological needs (17). On the other side, there is lots of evidence proving linking it’s use to stress, fear of missing out, and cybervictimization (18). The combination of unsupervised technology use, peer pressure, and the desire for social acceptance has resulted in excessive screen time on online dating apps, subsequently leading to concerning outcomes such as heightened risks for physical health and dating violence (19).

Excessive screen time relates to decreased academic outcomes and poorer performance in the educational activities (20, 21). Interactive and educational content may have different impacts compared to passive consumption like watching TV (22). Despite this, research links exposure to educational content to a lower risk of mental health issues. This suggests that not all screen time is detrimental, but content specifically designed for children can be beneficial (23).

Given the conflicting results of studies on the impact of social media on well-being, there is currently no consensus. The proposed Goldilocks’ hypothesis could provide a solution, as evidence suggests that moderate use of digital technology is not inherently harmful and may even have benefits (24, 25).

Adolescents (13–18) are not only heavy users of social media, but they are also at a crucial stage of development—a new stage of socialization and identity formation, making them more vulnerable to the effects of social media use and extensive screen time. Most teenagers have free access to mobile gadgets and social media from 13 to 15, which significantly raises their daily screen time (26). Their involvement shifts from passive consumption, shown by YouTube, to active, socially motivated interactions, as seen by TikHub and Instagram DMs (27). In contrast to young adults, who often engage with social media for networking or career objectives, teens primarily employ it for social interaction and self-presentation (28). Despite not possessing the full cognitive maturity of young adults, teenagers have greater flexibility in gadget usage compared to pre-adolescents, hence increasing their susceptibility to excessive engagement and the proliferation of social media addiction (9). Based on the effects of social media content on various outcomes, such as mental health and academic performance, along with the distinctiveness of the digital environment, adolescents represent a critical population for research and intervention.

### 1.2 Adolescent’s use of social media in Cuba and Russia

The digitalization processes are unique for Cuba due to the technological and economic reasons resulting in the special digital background (29). The past limitation of mobile data usage and the current steady growth of Internet use make Cuba a unique case to analyze the impact of screen time on physical and mental well-being. Nowadays, about 40% of adolescents and emerging adults’ social media takes up to 4 h a day, with a prevalence of messaging and commenting on the posts. Cuban youth and emerging adults tend to use social media not only as a tool for sharing entertainment related content but also a lot of personal information, but the tendency to excessive use leads to family issues and creates flaws for face-to-face interactions (29). To prevent the occurrence of the different social media related problems the Cuban university creates the social media addiction prevention program to foster the responsible use of social networks (30). Despite the uniqueness of social media consumption among Cuban youth, there are some common features that are revealed in the preferred content, motives of social network use, and relations to the information presented on the Internet. The recent study on the sample of 3,345 Cuban youth showed that social networks play a significant role in personal interactions, meeting the affiliation motive, the personal growth motive, and the self-presentation motive. Researchers found a two-way relationship between digital socialization and leisure time, which is mediated by the mental models of spare time and time spent on social media (31).

The research conducted on Russian adolescents indicated a rise in screen time, as measured by applications within the smartphone operating system, from 4 h at age 12 to 6 h at age 15, with a notable emphasis on messaging and video applications. In addition, screen time revealed as a significant negative predictor for the achievement motivation for teenagers aged 16–18 (32). Russian adolescents have different profiles of personality traits and online behavior, but two distinct groups could be divided—the group with a dominant orientation toward maintaining social relations and the group with an orientation to belongness and self-presentation (33). Another study categorizes the reasons for social media use among Russian youth into entertainment and productive motives, which can be viewed as endpoints of a continuum (34). Teenagers between the ages of 14 and 17 spend approximately 8 h on social media, primarily for the purpose of interacting and making new contacts, which can lead to an increased risk of cyberbullying. The study examining online behavior features as predictors of psychological well-being revealed that the integration of social media into daily life is associated with a decrease in risk, while the breadth of contacts and posting activity contributes to increased life satisfaction (29). The recent study of Russian teenagers revealed gender differences. Girls significantly better integrate social media into everyday life activities, have more online friends, and spend more time online. However, girls are more inclined to feel loneliness (35).

### 1.3 Current study

Despite the extensive literature on the impact of screen time, there is a gap in research regarding how these effects vary across different sociocultural contexts. Most studies have focused on adolescents in Western countries such as the United States and Europe, leaving in the background the analysis of populations with varying levels of access to technology and distinct cultural frameworks. The comparison between Cuba and Russia offers a unique opportunity to explore how factors such as digital infrastructure, internet access regulation, and family norms influence the effects of screen time. The uniqueness of the Cuban case lies in the historical restrictions on internet access and the recent expansion of its digitalization, which could generate consumption patterns distinct from those observed in countries with a longer history of connectivity (29). In contrast, Russia presents a more developed digital environment, with greater penetration of social networks and a digital culture closer to that of other European nations (34). This comparison can provide valuable insights into how structural and cultural conditions mediate the relationship between screen time and adolescent well-being.

The current study aimed to compare the screen time and online activity and its relationship with the psychological well-being between Cuban and Russian adolescent participants. This study is partly exploratory, as the previous results of such a comparison are limited. So, the following research questions were raised for that study:

RQ1: In what characteristics of screen time, social networks, addiction, well-being, and problems caused by the Internet and social media, and preferred content are there differences between Russian and Cuban participants?

RQ2: How do screen time, social network addiction, excessive screen time-induced alterations, and psychological well-being variables correlate in accounting for the country of residence?

RQ3: How well can the set of variables, including well-being variables and Internet activity, predict screen time-induced alterations when accounting for socio-demographic characteristics?

## 2 Materials and methods

We measured *screen time* on the Internet, excluding online classes during workdays and weekends, using two self-reported questions, i.e., “*How many hours have you spent on the internet (not including online classes) during a regular weekday (schoolday) in the last couple of months?*” To indicate the amount of time spent on the Internet, the respondents selected one of the following options: (0) less than 1 h per day, (1) about 1–2 h per day, (2) about 2–4 h per day, (3) about 4–6 h per day, or (4) more than 6 h per day.

The survey included a question about *the preferred content for interaction or posting*. We used the questions *(“How often do you interact or post with this type of content in the social media during the last couple of months?”)* adapted from the previous study of Cuban youth (29). Respondents were asked to indicate the frequency of interaction for each of the five theme categories: Trends and Inspiration, Education, Humor, Private Life, and Policy and Society. A Likert-type scale was employed, with response options spanning from never (0) to very often (4). Various *social media-related problems* associated with social media use were measured using symptoms representing four groups: screen fog (anxiety when using social media is limited and lowering of attention and concentration), vital drift (problems sleeping and changing eating habits), social withdrawal (being alone and not having face-to-face interactions with other people), and family disconnection (family members being aware of a lack of connection). We asked the respondents to answer the question “*Have you felt that some of these situations have happened to you in the last couple of months, following the use of social media?*,” and rate how frequently each of the symptoms appeared. We use the Likert-type scale, which ranges from never (0) to very often (4), for evaluation. The composite measure, which averages all indicators, was counted for overall screen-time-induced alterations. These questions were taken from the study concerning the social media use of Cuban young people (29).

To reduce the chance of recall bias, we used questions about screen time and social media from studies that showed these self-reported questions are fairly reliable (36, 37). Additionally, we included time references and context clues as suggested ways to match self-reports with actual usage data (22, 38). In addition, we used temporal anchors and contextual prompts as recommended methods to align self-reports with objective usage data (22, 38).

Positive and Negative Affect Schedule (PANAS) was used for general well-being evaluation. The PANAS is a reliable and valid instrument that consists of two scales designed to measure positive (PA) and negative (NA) affects (39). Each scale includes 10 words that describe a series of feelings and emotions. Respondents are asked to indicate the extent to which they usually feel them using a Likert-type scale ranging from very slightly or not at all (1) to extremely (5). The present study used the Russian version, adapted by Osin (40), and the Spanish version, adapted by Ortuño-Sierra et al. (41). For the Cuban sample, Cronbach’s alphas are 0.89 for the PA and 0.82 for the NA; for the Russian sample, they are 0.89 for the PA and 0.88 for the NA. Total scores on each scale (PA and NA) are obtained by adding the scores for each item.

Excessive social media use was assessed with the Bergen Social Media Addiction Scale (BSMAS). The scale was developed by Andreassen et al. (9) and comprises six items based on the six core components of social media addiction. Using a five-point Likert-type scale ranging between 1 (very rarely) and 5 (very often), respondents were asked to examine their experience of using social media over the past year. A higher score in the BSMAS indicates a greater likelihood of being at risk of developing a social media addiction. The appropriately adapted versions were used for Russian (42) and Spanish-speaking participants (55). Internal consistency (Cronbach’s alpha) of the scale is 0.79 and 0.06 for Russian and Cuban samples, respectively.

The scales and questions were presented online in one survey, which took approximately 30 min. The survey was conducted during off-class time at school. Participants received the survey link from a teacher or school psychologist who had been previously informed by the researchers about the study and could assist them during the survey process.

### 2.1 Sample

The Cuban participants were recruited from educational institutions that have working agreements with the Faculty of Psychology at Havana University. The Russian participants were recruited in the same manner from an educational institution that collaborates with the Federal Scientific Center of Psychological and Multidisciplinary Research. The study exclusively examined secondary education institutions, comprising conventional high schools. No schools are excluded from the selection process. This method made it easier to recruit participants while still being careful about how the study was done, allowing for comparisons between countries but also acknowledging that the results might not apply to a wider group.

The study initially recruited a total sample of 624 participants, consisting of 343 Russian and 281 Cuban participants, all of whom successfully completed the survey. The exclusion criterion (personal ownership of the device and access to the device at any time) was established to avoid bias due to the unequal access to devices. Thus, the study sample consisted of 300 adolescents from Russia and 224 adolescents from Cuba, all of them live in the urban area.

The Russian sample ranged from 14 to 18 years of age, with an average age of 15.67 years (SD = 0.89), 35% male. The participants resided with their parents and can be considered part of a middle-class family in Moscow, which has the highest Human Development Index in Russia (43). Most participants (80%) had parents who held at least a university degree, while 16% of parents had attained a college degree. The Cuban sample ranged from 13 to 18 years old, with an average age of 15.66 years (SD = 1.57), 46% male. The participants came from middle-class families with stable jobs and permanent residences in Cuba. Most of them lived in the municipality of Plaza de la Revolución of Havana, one of the areas with the highest Human Development Index in the city. 61% of participants had parents who held at least a university degree, while 30% of parents hadcompleted college.

Concerning the academic performance, the Russian sample (*N* = 300) had a mean GPA of 4.39 out of 5. In contrast, only a portion of the Cuban sample (*N* = 78) reported their mean academic index, which was 93.04 out of 100. In both educational systems, the obtained values can be considered high. Unfortunately, due to incomplete data from the Cuban sample and the reliance on self-reported GPAs for both samples, comparisons were potentially ineffective. In general, we could say that adolescents self-reported a high level of academic performance.

Adolescents participated in the study voluntarily, with written informed consent obtained from their parents or legal guardians. Informed consent was obtained offline from participants’ parents or legal guardians through the administration of the educational institutions, in accordance with ethical guidelines. The study received the approval from the ethics committee of the Federal Scientific Center of Psychological and Multidisciplinary Research (Russia), and the ethics committee the Faculty of Psychology at Havana University (Cuba), and was conducted in accordance with ethical guidelines and the principles of the Declaration of Helsinki, the Ethical Principles of Psychologists in Cuba, and the Russian Psychological Society. Participants were provided with a brief description of the study and anonymity guarantees.

All statistical analyses were conducted using the free, open-source, standalone software Jamovi (44). To analyze the differences between Cuban and Russian adolescents in the variables of interest, independent samples t-tests were performed for mean comparisons. Pearson correlation coefficients were calculated between well-being variables and screen time. Additionally, linear regression models were used to evaluate the impact of screen time and social media addiction on screen-induced disturbances, controlling for age and gender.

## 3 Results

### 3.1 Descriptive statistics

Primary the comparison of the age of the participants and male-to-female ratio of study samples was made. There is no significant differences in the age were found. A comparison of the male-to-female ratio indicated a notable predominance of women in the Russian sample (*χ*^2^ = 24.2, *df* = 1, *p* < 0.001). There is also a significant overwhelming of women in the Russian sample in comparison with Cuban sample (*χ*^2^ = 5.68, *df* = 1, *p* = 0.02; Cramer’s V = 0.10).

### 3.2 Differences in the study variables between Russian and Cuban adolescents

The comparison of the frequencies in screen time on weekdays and weekends between and within countries was made, Table 1. The chi-square test is significant for weekend screen time (*χ*^2^ = 11.7, *df* = 4, *p* = 0.02) and for the weekdays screen time (*χ*^2^ = 18.8, *df* = 4, *p* < 0.001), meaning there is a statistical association between nationality (Russia vs. Cuba) and internet use frequency. However, the Cramer’s V for weekend screen time (0.15), and weekdays screen time (0.19) suggests a small effect size—while the difference is statistically significant, the practical impact is not very strong. The chi-square test for within-sample differences in weekday screen time revealed significant differences in the proportion of time spent in online categories for both Russian adolescents (*χ*^2^ = 122, *df* = 4, *p* < 0.001) and Cuban adolescents (*χ*^2^ = 34.9, *df* = 4, *p* < 0.001). The same result was obtained for the weekend screen time — Russian sample (*χ*^2^ = 165, *df* = 4, *p* < 0.001) and Cuban sample (*χ*^2^ = 63.4, *df* = 4, *p* < 0.001).

**Table 1:** Time spent online percentage on weekdays and weekends among Cuban and Russian adolescents.

Time spent online	Weekdays			Weekend		
	Cuba	Russia	Total	Cuba	Russia	Total
Less than 1 h per day	5.8%	1.3%	3.2%	4.0%	2.3%	3.1%
About 1–2 h per day	17.0%	10.3%	13.2%	12.9%	5.7%	8.8%
About 2–4 h per day	26.3%	35.7%	31.7%	21.0%	18.7%	19.7%
About 4–6 h per day	24.6%	31.0%	28.2%	28.1%	33.0%	30.9%
More than 6 h per day	26.3%	21.7%	23.7%	33.9%	40.3%	37.6%

The differences in social media addiction, positive and negative effects, as well as social media-related problems indicators and preferred content for interaction or posting, were found using the t-test.

The significant differences revealed for most of the study variables with a little exception, means, and SDs in Table 2. Cuban participants have lower scores in the social media addiction (*t* = −5.70; *p* < 0.001; *d* = −0.50), and the effect size is moderate. Among the psychological well-being variables, there are no significant differences in positive affect, but Russian participants significantly overcome Cuban peers in negative affect (*t* = −4.22; *p* < 0.001; *d* = −0.37) with a relatively small effect size.

**Table 2:** Means and SDs for the study variables in the Cuban and Russian samples.

Variables	Cuban sample		Russian sample	
	Mean	SD	Mean	SD
Age	15.66	1.57	15.67	0.89
Time spent online on the weekdays	2.49	1.21	2.61	0.98
Time spent online on the weekend	2.75	1.17	3.03	1.01
Social media addiction	1.0	0.70	1.40	0.86
Positive affect	2.25	1.00	2.16	0.81
Negative affect	1.25	0.85	1.58	0.92
Vital drift	1.66	1.20	1.99	0.88
Social withdrawal	1.22	1.18	1.55	0.98
Screen fog	0.99	1.04	1.58	0.94
Family disconnect	1.25	1.40	0.86	1.04
Screen time-induced alterations	1.28	0.87	1.49	0.74
Trends and inspiration content	1.58	1.07	1.85	1.04
Educational content	0.98	0.96	1.71	1.09
Humorous content	2.25	1.38	2.38	1.33
Private life content	1.13	1.12	1.66	1.27
Policy and society content	0.39	0.65	0.72	0.88

The vital drift (*t* = −3.55; *p* < 0.001; *d* = −0.31), screen fog (*t* = −6.82; *p* < 0.001; *d* = −0.60), and social withdrawal (*t* = −3.43; *p* < 0.001; *d* = −0.30) indicators of problems caused by social media are significantly higher among Russian participants. However, the and family disconnection (*t* = 3.67; *p* < 0.01; *d* = 0.32) are higher among Cuban participants. The composite estimate of screen-time-induced alterations is significantly higher among Russian participants (*t* = −3.01; *p* = 0.003; *d* = −0.27). The effect size for induced social media-related problems indicators varies from low to moderate.

The differences in the preferred content among the participants from the compared countries were revealed for most of the topics except humor. The effect size for the trends and inspirational (*t* = −2.96; p = 0.003; *d* = −0.26), educational (*t* = −7.99; *p* < 0.001; *d* = −0.71) content is moderate, and for policy and society is low (*t* = −4.79; *p* < 0.001; *d* = −0.43). The private life content revealed a moderate effect size (*t* = −4.79; *p* < 0.001; *d* = −0.42). The trend is that Russian participants are more involved in the activity of following most of the topics or posting the related content.

*Post hoc* power analyses conducted using G*Power 3.1 (45) indicated 82–99% power to detect observed effects, consistently exceeding the conventional 80% threshold (46). The analyses revealed High-Power Findings (Power ≥ 90%) for social media addiction, screen fog, educational content preference, policy and society, and private life content. Moderate-Power Findings (80% ≤ Power < 90%) were identified for negative affect, vital drift, social withdrawal, and trends and inspirational content. All reported effects reached statistical significance at *p* < 0.05, demonstrating robust detection capabilities across measures while showing particularly strong sensitivity in the high-power domains.

### 3.3 Correlation of the study variables for the Cuban, and Russian adolescents

Next, we created a zero-ordered correlation among all study variables, applying the Holm-Bonferroni sequential correction to control familywise error rates across multiple comparisons (59). Power analyses confirmed adequate sensitivity for detecting medium effects across all correlations, with ≥80% power for medium effects (*r* ≥ 0.20) in the Cuban sample and ≥90% power for medium effects (*r* ≥ 0.20) in the Russian sample. The correlations between time spent on the Internet (weekdays and weekends) and social media addiction revealed the same pattern for the Russian and Cuban samples (weekdays: r_Cuba_ = 0.249; r_Russia_ = 0.208; *p* < 0.05; weekends: r_Cuba_ = 0.291; r_Russia_ = −0.282; *p* < 0.001). In addition, time spent online on the weekend positively related with screen time-induced alterations for Cuban participant (*r* = 0.222; *p* < 0.05), and with Negative affect (*r* = 0.206; *p* < 0.05) for the Russian participants. Most of the correlations between social media addiction and other variables are similar in both samples, as are the correlations between positive and negative effects, Table 3 presented most of the result.

**Table 3:** Correlation of the study variables for the Cuban adolescents.

Variables	Cuban sample			Russian sample		
	Social media addiction	Positive affect	Negative affect	Social media addiction	Positive affect	Negative affect
Positive affect	−0.002	–	–	−0.171	–	–
Negative affect	0.223*	0.054	–	0.425***	−0.298***	–
Vital drift	0.195	−0.016	0.339***	0.390***	−0.332***	0.429***
Social withdrawal	0.353***	−0.160	0.363***	0.325***	−0.460***	0.562***
Screen fog	0.378***	−0.128	0.305**	0.431***	−0.418***	0.604***
Family disconnect	0.374***	0.077	0.143	0.399***	−0.281***	0.328***
Screen time-induced alterations	0.452***	−0.067	0.390***	0.499***	−0.481***	0.619***
Trends and inspiration content	0.261***	0.143	0.157	0.306***	0.161	0.044
Educational content	0.092	0.051	0.121	0.057	0.105	0.059
Humorous content	0.148	0.012	0.093	0.159	0.059	0.068
Private life content	0.272**	−0.038	0.170	0.334***	0.112	0.143
Policy and society content	0.071	0.061	0.077	0.119	0.088	0.143

*p < 0.05, **p < 0.01, ***p < 0.001. Age found positive correlation with vital drift for Cuban sample (*r* = 0.219; *p* < 0.001), and negative for Russian sample (*r* = −0.120; *p* < 0.001). Regardless the country age negatively correlated with family disconnection (r_Cuba_ = −0.197; r_Russia_ = −0.135; *p* < 0.001). In addition, among Cuban participants age correlated with screen fog (*r* = 0.141; *p* < 0.05) and preference of educational content (*r* = 0.171; *p* < 0.05).

A series of regression analyses were made to analyze socio-demographics and psychological predictors of the screen time-induced alterations (Table 4). The indicators of screen time-induced alterations, social media addiction, and both positive and negative effects, in addition with the gender, age, and country of residence, were included in the regression analysis. *Post hoc* power analysis (G*Power 3.1) (45) for all regression models confirmed >99% sensitivity to detect effects at *α* = 0.001, given the observed large effect sizes and sample size (*N* = 524).

**Table 4:** Regression analysis results for the screen time-induced alterations features as criterions.

Predictor	Overall screen-time induced alterations		Social withdrawal		Vital drift		Screen fog		Family disconnect	
	*β*	SE	*β*	SE	*β*	SE	*β*	SE	*β*	SE
Intercept	0.84	0.37	0.81	0.54	−0.33	0.53	−0.55	0.48	3.43	0.65
Gender	−0.06	0.06	−0.01	0.08	−0.19***	0.08	0.02	0.08	−0.01	0.10
Age	0.01	0.02	0.01	0.03	0.13***	0.03	0.07*	0.03	−0.15***	0.04
Country	−0.03	0.06	0.02	0.08	0.05	0.08	0.15***	0.07	−0.26***	0.10
Weekdays	0.04	0.03	0.01	0.04	0.04	0.04	−0.01	0.03	0.07	0.05
Social media addiction	0.31***	0.04	0.18***	0.06	0.14***	0.06	0.27***	0.05	0.31***	0.07
Positive affect	−0.18***	0.04	−0.23***	0.04	−0.10*	0.04	−0.20***	0.04	−0.03	0.05
Negative affect	0.37***	0.03	0.38***	0.05	0.28***	0.05	0.35***	0.04	0.11**	0.06

*p < 0.05, **p < 0.01, ***p < 0.001. Dichotomous variables were coded as follows: gender: female = 0, male = 1; country: Cuba = 0, Russia = 1. Overall screen-time induced alterations *R*2 = 0.41, *F*(7, 516) = 51.0, *p* < 0.001. Vital drift *R*2 = 0.25, *F*(7, 516) = 24.5, *p* < 0.001. Social withdrawal *R*2 = 0.32, *F*(7, 516) = 34.6, *p* < 0.001. Screen fog *R*2 = 0.39, *F*(7, 516) = 46.4, *p* < 0.001. Family disconnect *R*2 = 0.20, *F*(7, 516) = 18.5, *p* < 0.001.

The regression explained 41% of the variation in changes caused by screen time overall, with five significant predictors. Both affects are significant predictors with opposite impacts in addition to social media addiction. The negative affect along with social media addiction increase, while positive affect decreases the overall screen-time-induced alterations.

The set of predictors, including positive and negative affects and social media addiction, was revealed as significant for social withdrawal, with 32% of the explained variance. Socio-demographic variables have no significant effect on increased social isolation and limitation of face-to-face contacts.

The vital drift regression model explains 25% of the variation by the set of predictors including positive and negative affects, social media addiction as significant predictors. Gender and age also have a significant effect. Positive affect decreases, but negative affect, in conjunction with social media addiction, intensifies issues with sleep and eating habits. This effect is stronger for girls and older participants. In addition to the regression the *t*-test’s results support the gender differences in the vital drift encompassing sleep and nutritional issues [*t* (522) = 6.75, *p* < 0.001, Cohen’s *d* = 0.60].

Positive and negative affects, and social media addiction, accompanied by age and country of residence, showed as significant predictors for the screen fog, accounting for 39% of variability. The affects make the opposite impact on the screen fog; social media addiction increases the feelings of anxiety and anguish when social media is restricted. The older participants, primarily Russian residents, demonstrated these effects.

The regression model for Family Disconnect explains 20% of variability with the set of predictors comprising negative affect, social media addiction, age, and country of residence. The negative effect of coupling with social media addiction is increasing family complaints about the loss of connection. This effect is more pronounced for younger participants and the inhabitants of Cuba.

## 4 Discussion

### 4.1 The differences between Russian and Cuban adolescents in the internet activity, social networks addiction, and preferred content

The frequency of the screen time of Russian and Cuban samples differs. Russian adolescent participants, in comparison with Cuban peers, use the Internet more on weekdays, from 2 to 6 h and from 4 to more than 6 h on the weekends. This result corroborates with the facts that Russian participants spend the largest amount of time online on weekdays in comparison with most of the teenagers from European countries (34). Despite the significant differences in frequency of time spent online between Russian and Cuban adolescents, there is a general trend for Internet use on weekdays and weekends that remains consistent across countries. About 25% (on weekdays) and 40% (on weekends) of adolescents use the Internet for more than 6 h; around 30% use it for 4 to 6 h regardless of the day of the week; and around 30% (on weekdays) and 20% (on weekends) use it for 2 to 4 h. There may be other factors (e.g., socioeconomic or psychological) that contribute to internet use patterns more.

Generalizing the obtained differences in the studied variables, it could be admitted the following: Russian participants, in comparison with Cuban peers, are prone to be more social media dependent. The fact about Russian participants is in line with the previous study (34). Cuban participants’ family members express greater concerns regarding their disconnection and absence from family interactions (29).

The variety of topics provided by social media captivates Russian participants more, and it could support the fact that, for them, social media is the main source of information and interaction (47). In contrast, Cuban participants have fewer interests in different social media topics. A previous study about the content preferred by Cuban participants for posting showed that the trending and inspirational content (including music, art, fashion, and sport) was popular among two-thirds of participants (29).

The disparities between Russian and Cuban participants in terms of internet use, social media dependency, and content preferences can be understood through broader cultural and structural factors. Russian adolescent participants tend to spend more time online and experience greater emotional distress when they cannot access social media. This fosters greater involvement on social media as a primary means of information and entertainment (47). This aligns with research suggesting that digital dependence is higher in societies with unrestricted internet access, where online interactions become central to identity formation and peer validation (35). In contrast, Cuba’s limited and often expensive internet access creates an environment where adolescents must prioritize their online activities, leading to more purpose-driven digital consumption, particularly with educational content (29).

Russian adolescent participants exhibit lower level of family disconnection due to screen time, which aligns with studies showing that individualistic cultures tend to place greater emphasis on digital autonomy (34). The greater reliance on online platforms for socialization may contribute to a weaker emphasis on direct family interactions. Cuban families, on the other hand, maintain stronger expectations for face-to-face communication, which may explain why excessive screen time is perceived as a disruptive factor in family dynamics (29).

### 4.2 The relation of the internet activity, social network addiction, screen-time induced alterations, and psychological well-being variables among Russian and Cuban adolescents

For the Russian and Cuban participants, time spent on the Internet (regardless of the day of the week) was positively associated with social media addiction which is in line with other studies (57). Note that only Russian participants showed a negative correlation between negative affect and weekend Internet time spending. That fact partially relates to a previous study of the negative impact of screen time on the life satisfaction and sense of loneliness of Russian teenagers (35). Cuban adolescent participants demonstrate association between increased screen time on the weekends with screen time-induced alterations.

The correlations between positive and negative effects have the same pattern for both study samples. In the Russian sample, positive and negative affect are related to all indicators of problems induced by social media screen time, but the correlations are in opposite directions and are all moderate. In the Cuban sample, only negative affect shows the same correlation pattern. Previous studies have indicated that social media might positively increase the well-being; however, this effect is attributed not to the intrusive usage but rather to its role in socialization and self-development (17, 48).

All screen-time induced alterations positively relate to social media addiction as well as with the preference for private life content regardless of the country.

### 4.3 Predictors of the screen time induced alterations

Emphasizing general trends, the results of the regression analysis help to clarify the effects of social media addiction and emotional states on teenage well-being. Regardless of the country of residence, negative affects and social media addiction greatly impact many problems related to overuse of the internet—overall screen-time-induced alterations, social withdrawal, and neglect basic needs. In addition, positive affect appears to have a limited impact; it could be assumed that is rather a protective element that reduces different negative impacts. It could be speculated that maintaining a positive emotional state may help address some time-related issues; yet it might not be sufficient to counteract the stress that too frequent internet use causes on screen-time induced alterations.

These facts are in line with the studies that showed that interventions aimed at reducing problematic digital media use can improve mental health outcomes and reduce addictive behaviors in adolescents. Randomized controlled trials of online and app-based interventions (e.g., mindfulness, acceptance and commitment therapy, relationship-focused apps) have shown improvements in depressive symptoms, life satisfaction, and well-being among adolescents, especially those with higher initial avoidance or problematic use (49–51).

The effect of age reveals issues related to sleep, nutrition, and attention and concentration difficulties among older adolescents. In contrast, excessive social media screen time for younger adolescents is linked to increased family tension, resulting in more parental complaints and domestic conflicts.

The connection between sleep problems and nutritional issues with the excessive social media use seems to be more pronounced for girls. These facts support previous studies that demonstrate a more pronounced detrimental effect of excessive screen time on the decrease in physical activity and basic needs among girls (3, 52). Furthermore, older girls are more prone to neglecting basic needs, such as adequate sleep and nutrition, as their social media usage escalates. A prior study of Hispanic adolescents indicated that among girls, increased social media use was associated with decreased activity linked to lower self-reported psychological well-being and social support (53).

The cultural variation exhibits in the elevation of anxiety, the lowering of attention and concentration, which are higher for the Russian adolescent participants, and the familial disconnection, which is higher for their Cuban peers. Negative affect, coupled with social media addiction, exacerbates attention and concentration difficulties and emotional distress stemming from active social media use, particularly for older adolescents and Russian residents. Conversely, Cuban participants tend to experience more family-related issues due to excessive social media use combined with negative affect. These findings imply that excessive engagement in social media, combined with negative affect, plays a more significant role than mere screen time in predicting adverse psychosocial outcomes.

## 5 Conclusion

Cultural differences emerged as significant factors affecting the consequences of excessive internet use. Russian participants showed a higher inclination for extended online activity, which matched a development in some cognitive problems (decreased attention, lack of concentration) connected to social media use. This implies that in the Russian setting, internet use might be more absorbing and consuming, thereby perhaps replacing offline activities to a greater extent. Cuban participants reported notable family-related consequences stemming from excessive internet use, including heightened parental concerns and disruptions in family communication. This suggests that family relationships are different depending on culture. For example, Cuban families value closeness and direct communication, which makes the effects of too much digital use at home stronger. The results highlight the numerous and complicated effects of social media on teenage well-being. While negative affect and social media addiction are major predictors of many digital-related problems, individual variables including gender, age, and cultural background are quite important in deciding particular results.

The following general recommendations could be implemented to mitigate the detrimental effects of excessive screen time. Parents might provide explicit, culturally appropriate guidelines to offset the harmful consequences of teenage screen use. Parents could restrict recreational use for Russian adolescents to 2 h daily, incorporating breaks to prevent a decline in attention and concentration (34). Cuban adolescents’ parents could establish device-free family times to enhance interpersonal communication. They can model healthy screen habits by avoiding phones during meals and co-viewing content, which encourages family engagement (54, 56). In Cuba, school programs could focus on responsible social media use to balance academic and leisure activities (30); in Russia, given the greater emphasis on digital interactions, educators should integrate critical digital literacy programs addressing social media dependency, online misinformation, and anxiety management (48).

The first limitation for this study is its cross-sectional nature, which provides only correlational results; however, it’s the first comparative study of screen time between Cubans and Russians, and, and it could be the initial point for further research. The second limitation is the sample characteristics, which were more convenient than strictly equalized. As noted in the sample description, participants were recruited from educational institutions that collaborate with the research team’s institutions. The socioeconomic backgrounds of the adolescents from the two countries were not directly compared, which limits the generalizability of the findings. However, it is important to recognize that all participants resided in capital cities and came from middle-class families. Finally, the evaluation of screen time relied on self-reported data; therefore, further studies should employ cross-check methods to validate the self-reported information and enhance the validity of the results.

## Data Availability

The raw data supporting the conclusions of this article will be made available by the authors, without undue reservation.
